# Use of a Remote Eye-Tracker for the Analysis of Gaze during Treadmill Walking and Visual Stimuli Exposition

**DOI:** 10.1155/2016/2696723

**Published:** 2016-01-19

**Authors:** V. Serchi, A. Peruzzi, A. Cereatti, U. Della Croce

**Affiliations:** ^1^Information Engineer Unit, POLCOMING Department, University of Sassari, Viale Mancini 5, 07100 Sassari, Italy; ^2^Interuniversity Centre of Bioengineering of the Human Neuromusculoskeletal System, Sassari, Italy

## Abstract

The knowledge of the visual strategies adopted while walking in cognitively engaging environments is extremely valuable. Analyzing gaze when a treadmill and a virtual reality environment are used as motor rehabilitation tools is therefore critical. Being completely unobtrusive, remote eye-trackers are the most appropriate way to measure the point of gaze. Still, the point of gaze measurements are affected by experimental conditions such as head range of motion and visual stimuli. This study assesses the usability limits and measurement reliability of a remote eye-tracker during treadmill walking while visual stimuli are projected. During treadmill walking, the head remained within the remote eye-tracker workspace. Generally, the quality of the point of gaze measurements declined as the distance from the remote eye-tracker increased and data loss occurred for large gaze angles. The stimulus location (a dot-target) did not influence the point of gaze accuracy, precision, and trackability during both standing and walking. Similar results were obtained when the dot-target was replaced by a static or moving 2D target and “region of interest” analysis was applied. These findings foster the feasibility of the use of a remote eye-tracker for the analysis of gaze during treadmill walking in virtual reality environments.

## 1. Introduction

Visual sampling may play a crucial role during challenging locomotor tasks [1] and previous studies involving obstacle avoidance showed that effective visual behavior is important for safe locomotion [2–4]. Rehabilitation programs including motor and cognitive aspects (e.g., obstacle negotiation exercises [5–7]) should assess both motor and visual strategies [8, 9]. However, this is rarely the case, most probably due to the complexity of the experimental setup that is required. In fact, the validity of an experimental study aiming at measuring gait and gaze while moving in a complex environment recreated in a laboratory setting may be challenged by the difficulty of designing tasks similar to those performed in real life [10]. The use of virtual reality (VR) environments allows for the most part to overcome such limitations and to create safe, repeatable, and controlled experimental setups. Furthermore, the integration of VR, gait analysis, and eye-tracking allows for a full control of the environmental variables while evaluating the subject's performance in terms of visual and locomotion variables. In this context, projected VR environments have been successfully used to elicit visual behavior similar to those observed in a real environment [10]. Moreover monitor-based projected VR has been successfully employed in several gait rehabilitation protocols and its feasibility and acceptance have been tested for several pathologic populations [5–7].

The point of gaze (PoG) can be measured using either wearable or remote eye-trackers (rET) [11, 12]. Recent literature evidenced that rETs should be preferred to wearable eye-trackers since they allow for unobtrusive tracking of the gaze and, hence, for more natural head movements and longer periods of data collection [12, 13]. Modern rETs permit recording gaze within a limited volume of operation even if the head is not completely stationary [12, 14]. The use of a treadmill and a projected VR environment represents a convenient technological solution to analyze gait kinematics while recording PoG. In fact, this setup allows limiting the volume in which the head motion is measured and maintaining the relative distances and angles between the subject, the rET, and the visual stimulus source within predefined ranges.

In general, the quality of the PoG measurements depends on the specific rET characteristics (i.e., camera resolution, sampling frequency, pupil illumination mode, and binocular or monocular vision mode). The reliability of the PoG measurements is also influenced by several factors independent of the rET characteristics. Some of them are subject specific (the morphology and physiology of the subject's eyes); others depend on the experimental conditions (the operator's expertise in calibrating the rET, changes in the environmental light and in the brightness of the stimulus, environmental interferences, and the range of motion of the subject's head) [12, 15]. Moreover, accuracy and precision of the PoG are crucial for interpreting the collected data, particularly when areas defined around a stimulus (regions of interest, RoI) are used to classify the visual behavior [12, 16].

Unfortunately, while few studies have investigated the influence of some of the above-mentioned critical factors on the PoG quality [16–19], to the authors' knowledge none of them have explored the use of rETs during a dynamic motor task such as walking. The goal of this study was to evaluate the appropriateness of a rET (Tobii TX300), in an experimental setup requiring the subject to walk on a treadmill while looking at projected targets. Good practice guidelines, which can be extended to more complex experimental conditions and visuomotor rehabilitation protocols, are also provided. The following critical factors were investigated: (a) definition of the rET workspace; (b) evaluation of the spatial accuracy, precision, and trackability of the PoG measurements for different locations of the stimuli while either walking or standing; and (c) applicability of RoI analysis to rET PoG measurements while walking.

## 2. Materials and Methods

### 2.1. Participants

Ten healthy subjects (Caucasian, 5 m., 5 f., height: 1.7 ± 0.1 m; age: 36.3 ± 9.5 y. o.) not wearing glasses nor contact lenses volunteered to the study.

### 2.2. Experimental Setup

**Figure 1:**
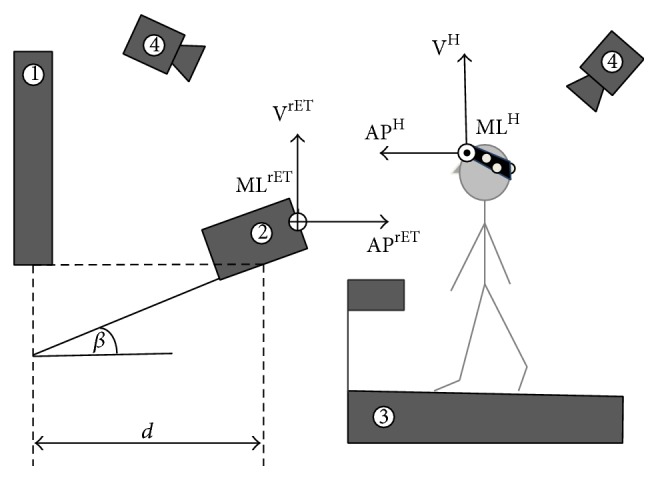
A schematic representation of the experimental setup, which included a screen (1), a rET (2), a treadmill (3), and a stereophotogrammetric system (4). Three retroreflective markers were placed on the subject's head to track its movements. The inclination of the rET with respect to the horizontal plane (*β*) and its distance from the projecting surface (*d*) were, respectively, set to 18 deg and 690 mm. The head reference frame and the rET reference frame are reported.

The experimental setup consisted of a treadmill, a rET (Tobii TX300, sampling at 300 frames/s), a projector (Epson, WXGA), a screen, and a marker-based 6-camera stereophotogrammetric system (Vicon T20, sampling at 300 frames/s). The treadmill, the rET, and the projecting surface were arranged as shown in Figure 1. The cameras of the stereophotogrammetric system were positioned to limit the infrared interference with the rET and care was paid that none of the cameras faced neither the subject's eyes nor the rET sensor [20].

The image on the screen was of 1040 mm × 580 mm (1280 px × 1024 px). The height of both the projector and the rET was adjusted for each subject. The center of the image was set at the same height of the subject's eyes, whereas the rET was placed at the same height of the lower edge of the image. Five retroreflective markers were placed on the front of the rET (one in each corner and one over a point indicated in the user manual as the point to which all the configuration measurements are referred [20]). Four additional markers were placed over the corners of the image projected so that image position and orientation could be determined with respect to the rET. Finally, three retroreflective markers were attached on a headband worn by the subject. The headband was adjusted so that one marker was over the inion and another above the left ear of the subject. The stereophotogrammetric system and the rET were synchronized via a TTL pulse generated by the stereophotogrammetric system at the acquisition start [20–22].

### 2.3. Acquisition Protocol

A reference frame embedded with the rET was defined using the retroreflective markers attached to the rET: the origin was made to coincide with the center of the rET sensor, the vertical (V^rET^) axis pointing upwards, the anterior-posterior (AP^rET^) axis parallel to the floor and pointing toward the subject, and the mediolateral (ML^rET^) axis orthogonal to both V^rET^ and AP^rET^ (Figure 1). A static stereophotogrammetric acquisition was performed to locate the image in the rET reference frame. The markers on the image corners and on the rET were then removed.

To define an anatomical head reference frame, an* ad hoc* calibration procedure was carried out while the subject was standing on the treadmill, with the eyes closed, while wearing both the headband and a marker attached on each eyelid [23]. The anatomical head reference frame origin was positioned in the midpoint between the two eyes, the mediolateral (ML^H^) axis was the line passing through the eyes, the vertical (V^H^) axis was orthogonal to the plane identified by the position of the two eyes and the inion and pointing upwards, and the anterior-posterior (AP^H^) axis was orthogonal to both V^H^ and AP^H^. The markers on the eyelids were then removed.

The use of the rET requires a preliminary subject-specific calibration of the two components of the PoG. This was carried out, for each subject, via the rET proprietary software (nine-point procedure; Tobii Studio, firmware 3.2, distance rET-subject equal to 650 mm). A calibration check was performed through the rET proprietary software which consisted in the generation of a circle around each calibration point within which a green dot was displayed in the case of a good calibration [20].

To characterize the performance of the rET, three different experimental sessions were carried out in a dark room.

#### 2.3.1. Workspace Identification

**Figure 2:**
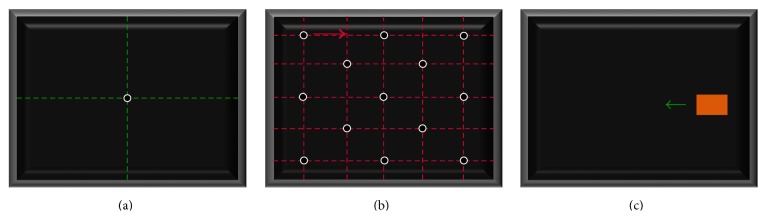
A schematic representation of (a) the visual stimulus used for the identification of the rET workspace; (b) the 13 dot-target locations of the visual stimulus on the screen used for the determination of the rET accuracy and precision; (c) the 2D target used to test the rET applicability for RoI analysis.

The subject initially stood on the treadmill facing the screen at a distance of 650 mm from the rET. The subject was asked to look at a dot-target located at the center of the image on the screen (Figure 2(a)), while translating anteroposteriorly (tAP, ~±200 mm), mediolaterally (tML, ~±100 mm), and vertically (tV, ~±100 mm) [24] and rotating the head around both ML^H^ (rML, ~±50 deg) and V^H^ (rV, ~±50 deg) axes. This task was performed to define the range within which the head of the subject could move without eye-tracking interruption.

#### 2.3.2. Accuracy and Precision Determination

The subject was asked to look at a dot-target displayed sequentially in 13 fixed locations of the image on the screen (Figure 2(b)). The dot-target persisted in each location for two seconds. Recordings were first performed with the subject standing still at 550 mm (st550), 650 mm (st650), and 750 mm (st750) from the rET. Then, the subject was asked to perform the same gaze task while walking at two different speeds (wslow: 0.6 m/s, and wfast: 1.1 m/s). The subject was free to hold the treadmill bar for safety.

#### 2.3.3. rET Applicability for RoI Analysis

The subject was asked to look at a 2D target used as visual stimulus (a rectangular shape, Figure 2(c), 230 mm × 80 mm) initially located at the center of the image (stat*_r*). After five seconds, the 2D target moved along a horizontal line at constant speed (80 pixels/s) from the right to the left (horiz*_r*) and along a vertical line from the top to the bottom (vert*_r*). The subject performed the task while standing at 650 mm from the rET (stRoI) and while walking on the treadmill at 0.6 m/s (wslowRoI) and 1.1 m/s (wfastRoI). The subject was free to hold the treadmill bar for safety.

### 2.4. Data Analysis

Blinks, saccades, short gaze deviations, and signal flickering instances were extracted from the PoG horizontal and vertical components. A validity score, provided by the rET proprietary software, is associated with each sampled PoG (0: eye found with high confidence; 4: eye not found). PoG time series scoring 4 for both eyes and longer than 100 ms were marked as blinks [12, 15, 25]. Saccades were identified as those PoG time series with velocity greater than 300 deg/s, amplitude greater than 7 deg, and duration higher than 20 ms [12]. The PoG outliers were removed according to [12]. Moreover, PoG time series with a velocity greater than 1000 deg/s, being not compatible with any physiological eye movement, were classified as flickering [12]. The first 800 ms of the stimulus presentation time was not considered in the data processing to take into account the physiological delay between the stimulus appearance and the transfer of gaze on it [26].

The data processing for each experimental acquisition is described below.

#### 2.4.1. Workspace Identification

For each subject, the minimum and maximum linear and angular values of the head position reached during the head movements (tAP, tML, tV, rML, and rV) were estimated and the relevant ranges of motion (RoM) computed. Similarly, for each subject, the minimum and maximum linear and angular values of the head position within which the rET was able to track both eyes were computed and referred to as ranges of trackability (RoTs). To each head movement, the median values of both RoMs (mRoM) and RoTs (mRoT), computed across subjects, were obtained. The rET workspace was defined as the combination of the mRoTs along the different directions.

#### 2.4.2. Accuracy and Precision Determination

For each trial (st550, st650, st750, wslow, and wfast), the PoG accuracy was computed as the estimated PoG distance from the known position of the *i*th stimulus (*i* = 1,…, 13) averaged over the stimulus presentation time and subjects (*ɛ*_*i*_). Similarly, the PoG precision was computed as the standard deviation of the estimated PoG averaged over the *i*th stimulus presentation time and the subjects (*δ*_*i*_). For each analyzed trial, the overall PoG accuracy and precision (*ɛ*, *δ*) were computed as the average values of *ɛ*_*i*_ and *δ*_*i*_ over all dot-target locations.

For each trial (st550, st650, st750, wslow, and wfast), the overall index of trackability of the PoG was computed as the percentage ratio between the valid and the expected samples during the *i*th stimulus presentation time averaged over the subjects and the dot-target locations (*ɤ*). For wslow and wfast trials, the head RoMs were computed for each subject to verify that the head moved within the rET workspace.

#### 2.4.3. rET Applicability for RoI Analysis

A RoI was defined by adding a margin, equal to the *δ* value obtained in the wfast trial, to the 2D target. The percentage of the PoG hitting the RoI was computed over the 2D target presentation time (%stat*_r*, %horiz*_r*, and %vert*_r*).

### 2.5. Statistical Analysis

#### 2.5.1. Accuracy and Precision Determination

A Friedman test for nonnormal distribution was used to assess (a) if *ɛ*_*i*_ and *δ*_*i*_ were statistically different among the dot-target locations, (b) if *ɛ* and *δ* were significantly different among trials st550, st650, st750, wslow, and wfast, and (c) if *ɤ* was statistically different among trials st550, st650, st750, wslow, and wfast.

#### 2.5.2. rET Applicability for RoI Analysis

A Friedman test for nonnormal distribution was performed to assess (a) if %stat*_r*, %horiz*_r*, and %vert*_r* obtained for each 2D target motion (stat*_r*, horiz*_r* and vert*_r*) were significantly different and (b) if %stat*_r*, %horiz*_r*, and %vert*_r* obtained for each motor task (stRoI, wslowRoI, and wfastRoI) were significantly different.

The level of significance was set to 0.05 for all statistical analyses. Pairwise comparisons were performed using a Wilcoxon signed-ranked test with a Holm-Bonferroni correction (*α* = 0.05) for the significant findings. The effect size* r* was computed for the significantly different pairs.

## 3. Results

### 3.1. Workspace Identification

**Figure 3:**
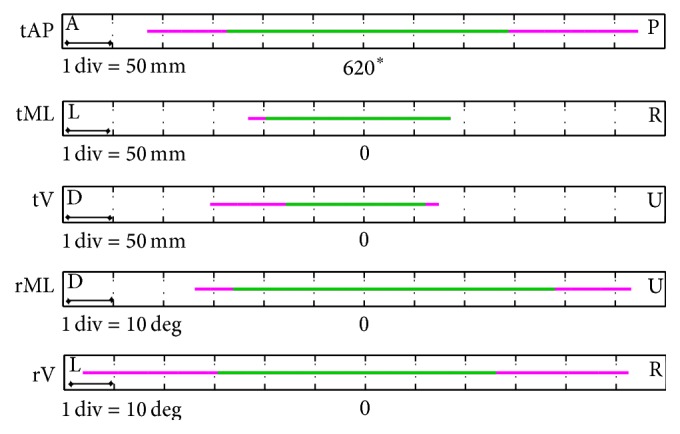
The head mRoTs (green) and mRoMs (magenta) along the AP^rET^, ML^rET^ (L, left; R, right), and V^rET^ (U, up; D, down) directions and around the ML^H^ (U; D) and V^H^ (L; R) directions. (*∗*) The tAP values are centered at 620 mm, which is the projection of the original distance from the sensor on the AP^rET^ axis (650 mm).

The rET mRoTs along and around the tested directions are shown in Figure 3 (translations: AP^rET^, 484 to 765 mm; ML^rET^, −98 to 86 mm; V^rET^, −78 to 61 mm; rotations: V^H^, −29 to 26 deg; ML^H^, −26 to 38 deg). No gaze tracking interruptions occurred for the positive translation along the ML direction.

### 3.2. Accuracy and Precision Determination

**Figure 4:**
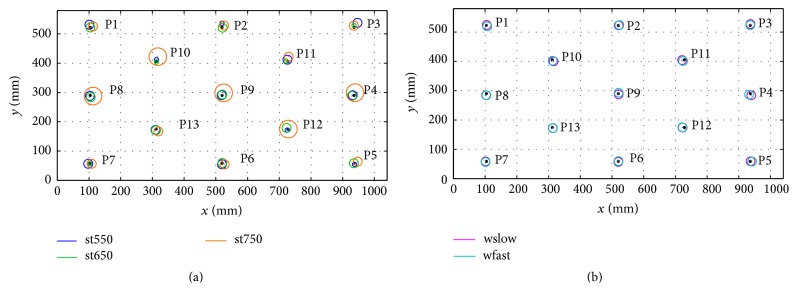
A graphical representation of *ɛ*_*i*_ and *δ*_*i*_ values found for each dot-target location during trials st550, st650, and st750 (a) and wslow and wfast (b). Each dot-target location on the image is a black dot. The circles center positions (colored dots) reflect the accuracy of the PoG measurements (*ɛ*_*i*_) while their radius reflects the precision of the PoG measurements (small radius, *δ*_*i*_ < 4 mm; average radius, 4 mm < *δ*_*i*_ < 8 mm; large radius, *δ*_*i*_ > 8 mm).

A graphical representation of *ɛ*_*i*_ and *δ*_*i*_ values found for each dot-target location during trials st550, st650, and st750 is reported in Figure 4(a). A similar graphical representation for the trials wslow and wfast is reported in Figure 4(b).

During trial st550, the PoG was lost in one of the top corners (P1 or P3) for three subjects. The values relative to these points in the trial st550 were excluded from the computation of the relevant following parameters. For *ɛ*_*i*_ and *δ*_*i*_ no significant differences were found among the 13 dot-target locations in any of the trials st550, st650, st750, wslow, and wfast.

The *ɛ*, *δ*, and *ɤ* values for st550, st650, st750, wslow, and wfast are reported in Table 1.

No significant differences were found among *ɛ* and *ɤ* values of the trials st550, st650, st750, wslow, and wfast. A significant difference was found among their *δ* values (*p* < 0.001). The value of *δ* computed for st750 resulted significantly larger than those computed for st550 (*p* = 0.040), st650 (*p* = 0.045), and wslow (*p* = 0.045) with a large effect size (*r* = 0.64, 0.63, and 0.64, resp.).

**Figure 5:**
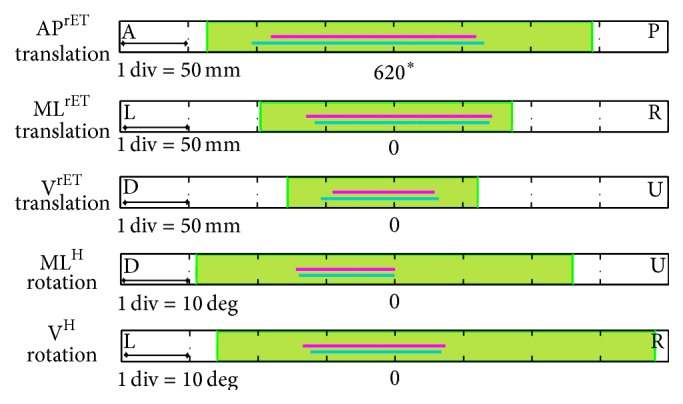
The intervals between the minimum and the maximum values of the head RoMs (green) obtained during the trials wslow (violet) and wfast (light blue) across the subjects: translations along the AP, ML (L, left; R, right), and V (U, up; D, down) directions and rotations around the ML (U; D) and V (L; R) directions. The green band represents the rET workspace. (*∗*) The values of the translations along the AP^rET^ direction are centered at 620 mm, which is the projection of the original distance from the sensor on the AP^rET^ axis (650 mm).

The intervals between the minimum and the maximum values of the head RoMs across subjects obtained during trials wslow and wfast are reported in Figure 5. The head motion remained always within the rET workspace.

### 3.3. rET Applicability for RoI Analysis

The values of %stat*_r*, %horiz*_r*, and %vert*_r* are reported in Table 2.

Neither the motion of the 2D target (stat*_r*, horiz*_r*, and vert*_r*) nor the motor task (stRoI, wslowRoI, and wfastRoI) significantly influenced the percentage of PoG (%stat*_r*, %horiz*_r*, and %vert*_r*) hitting the RoI.

## 4. Discussion

The main goal of the present study was to evaluate the suitability of the use of remote eye-tracking technology (Tobii TX300) during treadmill walking. This analysis can be relevant when developing projected VR-based applications aiming at investigating the visual behavior during walking in controlled environments. In particular, we aimed at defining the ranges of trackability, at providing a detailed description of PoG data quality during standing and walking (accuracy, precision, and trackability) and at testing the rET feasibility for dynamic RoI analysis. While previous studies [16, 17] limited the assessment of the PoG quality during various static head orientations and positions, we extended the rET testing under dynamic exercises such as gait.

The workspace identified in this study setup is in accordance with the datasheet of the device for what concerns the anterior-posterior head motion (500–800 mm distance from the rET), whereas the range of trackability along the mediolateral and vertical directions was slightly smaller than the nominal range declared by the manufacturer (mediolateral: ±100 mm; vertical: ±80 mm). In agreement with Blignaut and Wium [16], we found a general decline of the quality of the PoG measurements for larger distances from the rET (750 mm) and a few gaze losses for large gaze angles corresponding to the closest distance tested (in trial st550, a loss of PoG occurred on two dot-target locations located at the grid top corners). In another study, Hessels et al. [17] concluded that the quality of the PoG measurements is jeopardized at extreme head rotations around the V axis; however, no information about the amplitude of the angular head rotations was reported. Similarly to [17], we found some loss of PoG data during the head rotation around the vertical direction limiting the range of trackability to −29 to 26 deg.

The statistical analysis revealed that the position of the stimulus on the image does not influence the PoG accuracy, precision, and trackability both while standing and while walking. In particular, head displacements were within the ranges of trackability during walking trials (speeds up to 1.1 m/s), confirming that the Tobii TX300 can be conveniently used for the determination of the PoG during gait.

Furthermore, neither the motion of the 2D target (static, vertical, and horizontal) nor the motor task (static, slow, and fast gait) significantly influenced the percentage of the PoG samples hitting the RoI thus supporting the use of dynamic RoIs during the analysis of walking tasks. This finding fosters the usability of the rET TX300 for the gaze analysis during treadmill gait in projected VR-based applications. To the authors' knowledge, this study provides the first characterization of a rET used for tracking gaze while walking. This study represents also a fundamental preliminary step for a correct, unobtrusive assessment of the interactions between motor and visual strategies occurring during gait rehabilitation protocols requiring VR environments.

## 5. Conclusion

This study demonstrated that the rET TX300 can be used to analyze gaze during walking on a treadmill, since the performance of the rET and the quality of the measurements did not significantly differ from those obtained during static tasks. The outcomes of this study may provide elements for the design and implementation of analytical and experimental procedures for the combined analysis of gaze and human locomotion in VR-based applications.

## Figures and Tables

**Table 1:** PoG measurements accuracy *ε*, precision *δ*, and index of trackability *ɤ*. The values are averaged over the 10 subjects and over the 13 dot-target locations.

	st550	st650	st750	wslow	wfast
*ε* [mm]	13	10	17	12	13
*δ* [mm]	4	4	8	5	6
*ɤ* [%]	77	90	90	88	87

**Table 2:** The percentage of PoG hitting the RoI defined around the 2D target moving on the screen with different patterns (stat_*r*: static at the center of the screen; horiz_*r*: moving horizontally; and vert_*r*: moving vertically). Percentages are reported for each motor task: stRoI: standing at 650 mm from the rET; wslowRoI: walking at 0.6 m/s; and wfastRoI: walking at 1.1 m/s.

	%stat_*r*	%horiz_*r*	%vert_*r*
stRoI [%]	96 ± 3	98 ± 2	97 ± 4
wslowRoI [%]	96 ± 5	98 ± 2	94 ± 7
wfastRoI [%]	97 ± 3	98 ± 1	95 ± 6
